# Supplementary material for: Role of Histone Variant H2A.J in Fine-Tuning Chromatin Organization for the Establishment of Ionizing Radiation-Induced Senescence
Source: Cells. 2023 Mar 16;12(6):916. doi: 10.3390/cells12060916 (PMC10047397; doi:10.3390/cells12060916)
Supplement: Supplementary file 1 [file cells-12-00916-s001.zip › Suppl1 Volcano plots.pdf]

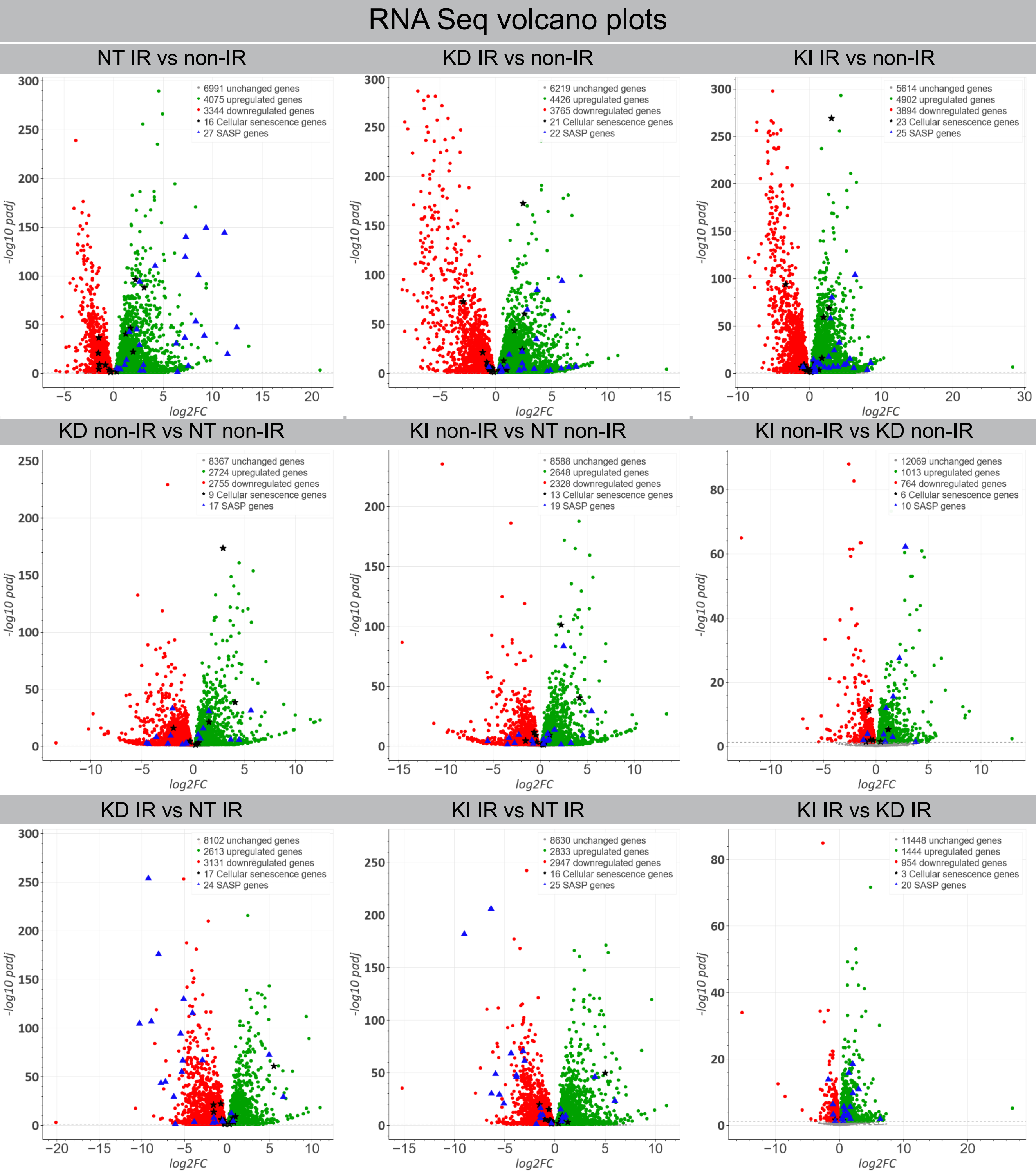
RNA Seq volcano plots
NT IR vs non-IR
6991 unchanged genes
4075 upregulated genes
3344 downregulated genes
16 Cellular senescence genes
27 SASP genes
KD IR vs non-IR
6219 unchanged genes
4426 upregulated genes
3765 downregulated genes
21 Cellular senescence genes
22 SASP genes
KI IR vs non-IR
5614 unchanged genes
4902 upregulated genes
3894 downregulated genes
23 Cellular senescence genes
25 SASP genes
KD non-IR vs NT non-IR
8367 unchanged genes
2724 upregulated genes
2755 downregulated genes
9 Cellular senescence genes
17 SASP genes
KI non-IR vs NT non-IR
8588 unchanged genes
2648 upregulated genes
2328 downregulated genes
13 Cellular senescence genes
19 SASP genes
KI non-IR vs KD non-IR
12069 unchanged genes
1013 upregulated genes
764 downregulated genes
6 Cellular senescence genes
10 SASP genes
KD IR vs NT IR
8102 unchanged genes
2613 upregulated genes
3131 downregulated genes
17 Cellular senescence genes
24 SASP genes
KI IR vs NT IR
8630 unchanged genes
2833 upregulated genes
2947 downregulated genes
16 Cellular senescence genes
25 SASP genes
KI IR vs KD IR
11448 unchanged genes
1444 upregulated genes
954 downregulated genes
3 Cellular senescence genes
20 SASP genes
-log10 padj
log2FC
