# Supplementary figures and images for: Role of Histone Variant H2A.J in Fine-Tuning Chromatin Organization for the Establishment of Ionizing Radiation-Induced Senescence
Source: Cells. 2023 Mar 16;12(6):916. doi: 10.3390/cells12060916 (PMC10047397; doi:10.3390/cells12060916)

# Histone expression ( RNA-seq)

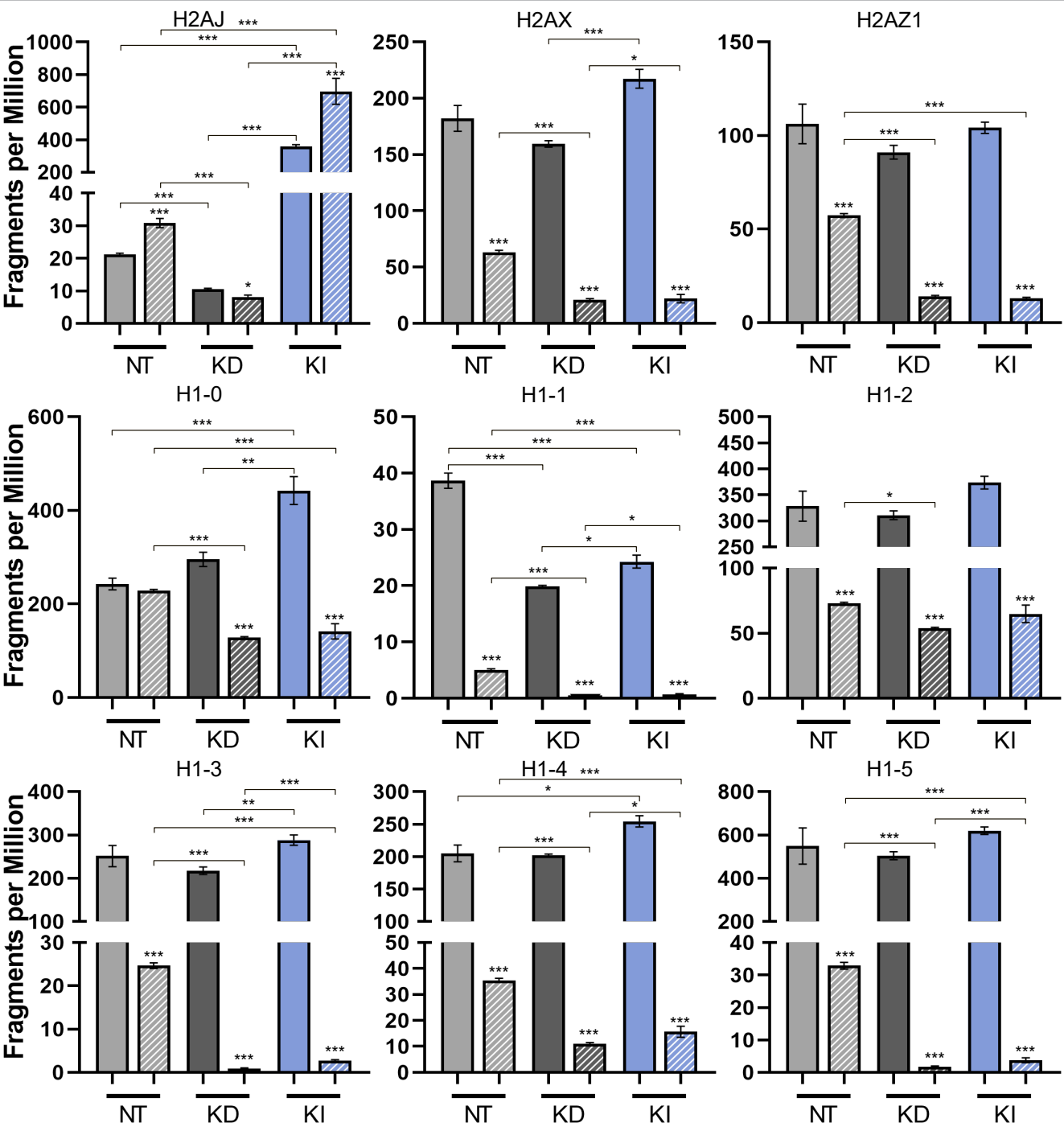

Supplement: Supplementary file 1 [file cells-12-00916-s001.zip › Suppl2 Histone expression part 1.pdf]

# Histone expression (RNA-seq)

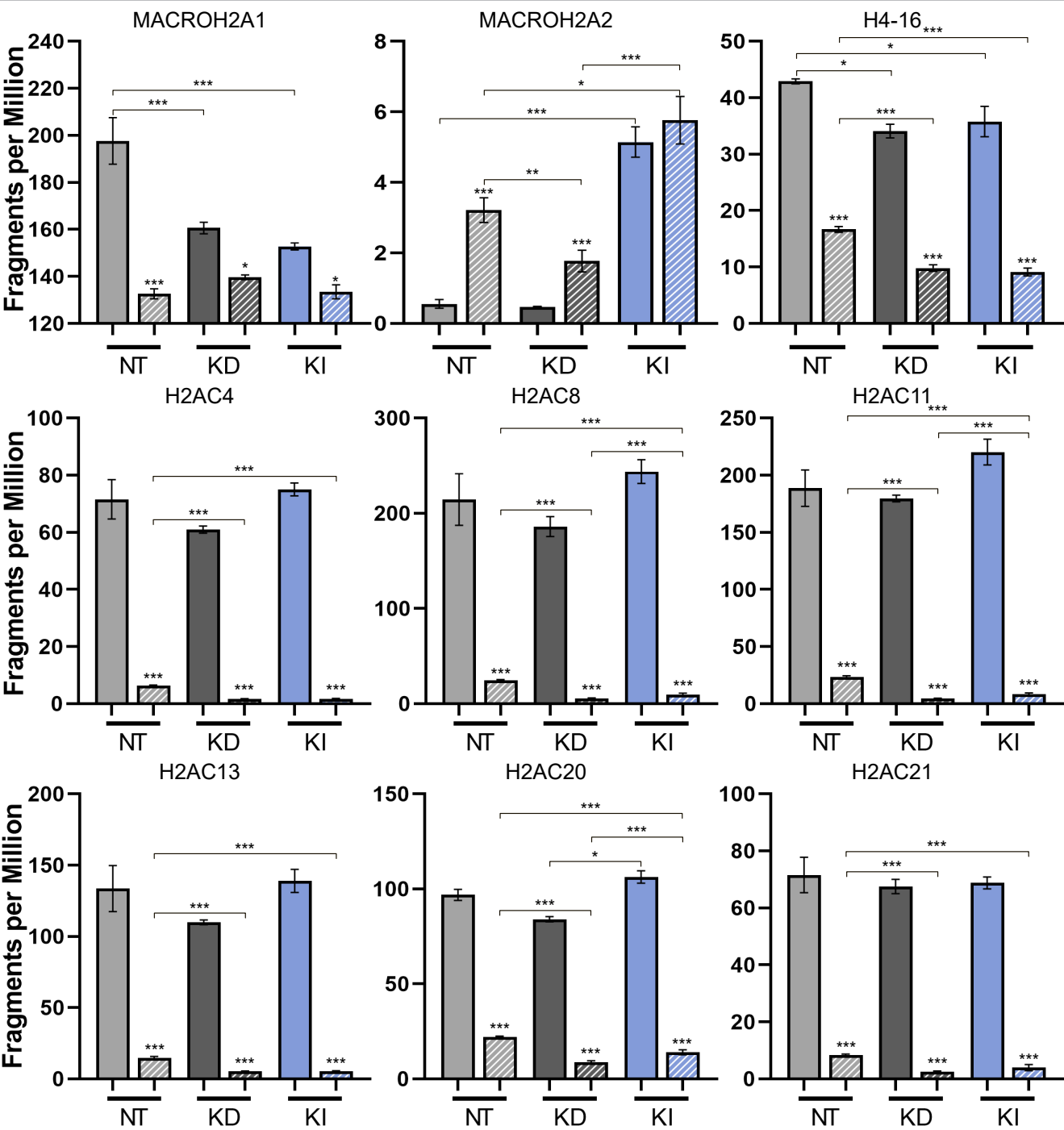

Supplement: Supplementary file 1 [file cells-12-00916-s001.zip › Suppl3 Histone expression part 2.pdf]
